# Identification of *ATRNL1* and *WNT9A* as novel key genes and drug candidates in hypertrophic cardiomyopathy: integrative bioinformatics and experimental validation

**DOI:** 10.3389/fmolb.2024.1458434

**Published:** 2024-09-12

**Authors:** Huabin He, Yanhui Liao, Yang Chen, Hao Qin, Longlong Hu, Shucai Xiao, Huijian Wang, Renqiang Yang

**Affiliations:** ^1^ Department of Cardiovascular Medicine, the Second Affiliated Hospital, Jiangxi Medical College, Nanchang University, Nanchang, China; ^2^ Department of Cardiovascular Medicine, Jiu jiang NO. 1 People’s Hospital, Jiujiang, China

**Keywords:** hypertrophic cardiomyopathy, bioinformatics, ATRNL1, Wnt9a, hsa-miR-1469

## Abstract

**Background:**

Hypertrophic cardiomyopathy (HCM) is a genetic disorder characterized by left ventricular hypertrophy that can lead to heart failure, arrhythmias, and sudden cardiac death. Despite extensive research, the molecular mechanisms underlying HCM are not fully understood, and effective treatments remain limited. By leveraging bioinformatics and experimental validation, this study aims to identify key genes and pathways involved in HCM, uncover novel drug candidates, and provide new insights into its pathogenesis and potential therapeutic strategies.

**Methods:**

Commonly upregulated and downregulated genes in hypertrophic cardiomyopathy (HCM) were identified using Gene Expression Omnibus (GEO) datasets, including three mRNA profiling datasets and one miRNA expression dataset. Enrichment analysis and hub-gene exploration were performed using interaction networks and consistent miRNA-mRNA matches. Potential drugs for HCM were screened. HCM cellular and animal models were established using isoproterenol. Key unstudied differentially expressed genes (DEGs) were validated. Animals were treated with novel potential drugs, and improvements in HCM were assessed via ultrasound metrics. Hematoxylin and eosin (H&E) staining was used to assess myocardial fibrosis. Immunohistochemistry was employed to detect DEGs in cellular experiments.

**Result:**

We discovered 145 key upregulated and 149 downregulated DEGs associated with HCM development, among which there are eight core upregulated and seven core downregulated genes. There are 30 upregulated and six downregulated miRNAs. Between the six downregulated miRNAs and 1291 matched miRNAs (against eight core upregulated DEGs), there is one common miRNA, miR-1469. Using the CTD database, drugs that impact the expression/abundance/methylation/metabolic process of core DEGs (after the exclusion of toxic drugs) included acetaminophen, propylthiouracil, methapyrilene, triptolide, tretinoin, etc. In the HCM cell model, only *ATRNL1* and *WNT9A* were significantly increased. In the HCM animal model, propylthiouracil, miR-1469, and triptolide demonstrated varying degrees of therapeutic effects on HCM. Propylthiouracil, but not miR-1469 or triptolide, significantly inhibited the expression of *ATRNL1* in the HCM model, and all three drugs suppressed *WNT9A* expression.

**Conclusion:**

We identified several novel genes in HCM development, among which *ATRNL1* and *WNT9A* were validated by cell and animal models. A deficiency of hsa-miR-1469 may be a mechanism behind HCM development. Novel medications for HCM treatment include propylthiouracil and triptolide.

## 1 Introduction

Hypertrophic cardiomyopathy (HCM) is an inherited cardiac disease that exhibits diverse genotypes, phenotypes, and clinical symptoms. It is the most frequently inherited cardiac disorder, with a prevalence of 0.2%–0.5% (Semsarian et al., 2015; Maron et al., 2022a). The typical symptoms are myocardial fiber hypertrophy, disarrangement, and myocardial hypertrophy, particularly asymmetric left ventricular hypertrophy. HCM usually results in obstruction of the left ventricular outflow tract (LVOT), which then triggers chest discomfort, dyspnea, fatigue, and syncope. The HCM phenotype can be observed in patients with varying degrees of left ventricular (LV) mass, ranging from mild LV wall thickness (≤15 mm) to massive hypertrophy (≥30 mm) (Maron et al., 2022b). Late diagnosis is very common, and HCM is a leading cause of sudden cardiac death, with an annual death rate of 0.5%–3% (Rowin et al., 2017; Maron et al., 2022c).

Currently, treatment options for HCM are limited. It is usually unnecessary to treat patients with obstructive HCM but no symptoms with medical or septal reduction therapy. However, these patients are still at risk, and there are no effective preventive medications available. Beta-blockers, such as nadolol and metoprolol succinate, are the preferred initial therapy for most patients with LVOT obstruction. A personalized decision is usually made about additional medical treatments, which encompass disopyramide combination therapy (e.g., disopyramide with a beta blocker or calcium channel blocker) or myosin inhibitor combination therapy (e.g., mavacamten with a beta-blocker or calcium channel blocker) (Olivotto et al., 2020; Tuohy et al., 2020).

Understanding the molecular mechanisms behind the pathogenesis of HCM can facilitate the development of effective treatment. It is widely acknowledged that genetic mutations play a key role in the occurrence of HCM. Based on the existing knowledge of the genetic and molecular basis of HCM and phenocopies (including myofilament mutation, Z-disc mutation, and calcium-handling), genes that are closely related to HCM include *MYBPC3*, *MYH7*, *TNNI3*, *TNNT2*, *TPM1*, *MYL2*, *ACTC*, *MYH6*, *LBD3*, *ACTN2*, *ANKRD1*, *JPH2*, and *PLN* (Geske et al., 2018). There is an insufficiency of information about validated and widely prevalent HCM genes in the existing research, causing a pressing need to explore potential drug targets and medications, for example, modulation of pathogenic/protective genes via known altered miRNAs or pharmacological interventions to assist in managing and preventing HCM.

In this study, the common upregulated and downregulated genes in HCM were identified using datasets from the Gene Expression Omnibus (GEO). Associated enrichment analysis was then performed, followed by hub-gene exploration through interaction network and screening of consistent miRNA-mRNA matches, as well as potential drugs for HCM. In addition, we validated key differential genes and novel therapeutic agents through cellular and animal models. The outcomes of this study may deepen the understanding of HCM development and improve the efficacy of its treatment in clinics.

## 2 Materials and methods

### 2.1 Data collection

We searched for the most recent transcriptome analysis data containing the HCM group and control group through the Gene Expression Omnibus (GEO) database (http://www.ncbi.nlm.nih.gov/geo/) using used the keyword “hypertrophic cardiomyopathy.” The following three mRNA datasets were used for one miRNA analysis. The organism was *Homo sapiens*, and datasets were acquired through the same or similar platforms. Additionally, the diagnostic criterion for HCM across all datasets is defined as follows: HCM in adults was determined by a wall thickness of ≥15 mm in one or more left ventricular (LV) myocardial segments, as measured by any imaging technique (echocardiography, cardiac magnetic resonance imaging, or computed tomography), which is not explained solely by loading conditions:

GSE206978: This set includes the data of transcriptome profiling and epigenome wide DNA methylation analysis.
GSE180313: This set surveys the altered cardiac energetics and mitochondrial dysfunction in HCM.
GSE160997: This set investigates the RNA-seq transcriptome profiling and whole exome sequencing data on HCM patients.
GSE197191: This set contains the microRNA (miRNA) profiling of HCM and control populations.

### 2.2 Differentially expressed genes

According to the grouping information, the sample expression data were imported into the DEGseq R package to compare the expression differences between samples. The fold change *p*-values were obtained. Then, differentially expressed genes (DEGs) were determined according to the threshold of *p*-value<0.05 and log2 (foldchange) > 1 or < −1. The volcano plots were produced to present DEGs using the ggplot2 R package. The Venn diagrams of DEGs in three different GEO datasets were drawn. The upregulated DEGs that appeared in all three sets were considered as the core upregulated DEGs, and the downregulated DEGs that appeared in all three sets were considered as the core downregulated DEGs. The upregulated or downregulated DEGs shared by any two sets were considered key DEGs in the following analysis.

### 2.3 Enrichment analysis

The Metascape online tool (http://metascape.org) was used for enrichment analysis based on the upregulated and downregulated key DEGs. All DEGs were imported into the tool, and pathway and process enrichment analysis were conducted with the following ontology sources: GO, KEGG Pathway, Reactome, Transcription Factor Targets, and DisGeNET sets. The *Homo sapiens* background was used for enrichment analysis of all DEGs. Terms with a *p*-value < 0.01, a minimum count of 3, and an enrichment factor > 1.5 (the ratio between the observed counts and the counts expected by chance) were collected. The statistically enriched terms (GO/KEGG/Reactome terms) were presented by accumulative hypergeometric *p*-values and enrichment factors. Significant terms were then hierarchically clustered into a tree based on kappa-statistical similarities among their gene memberships. A 0.3 kappa score was applied as the threshold to cast the tree into term clusters. Terms with a similarity score > 0.3 are linked by an edge, where the thickness of the edge represents the similarity score. The network was visualized with Cytoscape with the “force-directed” layout and edges bundled for clarity. One term from each cluster was selected to have its term description shown as a label.

### 2.4 Key gene interaction networks

Based on key upregulated and downregulated genes, the protein–protein interaction (PPI) network was constructed using the Search Tool for the Retrieval of Interacting Genes (STRING) database (http://www.string-db.org), which provides experimental and predicted protein interaction information. The organism was set as *H. sapiens*. The active interaction source included all terms except text-mining (experiments, databases, co-expression, neighborhood, gene fusion, and co-occurrence). The criterion of node connection was ≥ 0.4.

### 2.5 Consistent miRNA–mRNA matches

The miRWalk database was used for matched miRNAs of the core upregulated and downregulated mRNAs (Score = 1). The matched miRNAs of core upregulated mRNAs and the downregulated miRNAs in HCM (in the GSE197191 dataset) were analyzed, and the intersection was the set of consistently downregulated miRNA in HCM. Similarly, the interaction of miRNAs matching the downregulated mRNA and the upregulated miRNAs in HCM (in the GSE197191 dataset) was the consistently upregulated miRNAs.

### 2.6 Using the core DEGs to identify potential drugs for HCM

The CTD database (http://ctdbase.org) was used to probe for potential drugs that impact the expression of core DEGs. The upregulated and downregulated core DEGs were input, and the drugs that impact the expression/abundance/methylation/metabolic process of each gene were acquired. The drugs modulating the most targets (increasing the expression of most downregulated core DEGs or decreasing the expression of most upregulated core DEGs) were identified, and we paid special attention to drugs with no known toxicity that can be clinically used.

### 2.7 The HCM cell model

In brief, HL-1 cells (purchased from iCell-m077) were treated with 10 μM isoproterenol (ISO) for 24 h and 48 h to induce an *in vitro* HCM cell model. Before the formal modeling, cell culture was performed with complete DMEM/F12 medium (KGL1203-500, KGI Bio) at 37°C in a 5% CO_2_ incubator. HL-1 cells were first treated with vehicle, 0, 1 μM, 2.5 μM, 5 μM, 10 μM, 20 μM, 40 μM, and 80 μM ISO for 24 h. Then, the proliferation status of HL-1 cells at various ISO concentrations was assessed using the CCK8 assay to determine the optimal ISO treatment concentration. In addition, the phenotype of HCM was confirmed by phalloidin fluorescent staining (showing actin in the cytoskeleton). The cells in each well were fixed with a 4% fixative (P1110, Solarbio) for 15 min, washed three times with PBS, and permeabilized with 0.5% Triton X-100 at room temperature for 20 min. Next, cells were washed with PBS three times (5 min each time). Phalloidin (1:200, CA1610, Solarbio) was added, and cells were incubated at room temperature for 30 min and washed with PBS. DAPI was added and incubated for 3 min in the dark. Cells were washed with PBS and stabilized with 50% glycerol. The phalloidin staining was observed under a fluorescence microscope (CKX53, Olympus).

### 2.8 Validation of key gene expression in the HCM cell model

After concentration selection, HL-1 cells were treated with ISO for 24 h and 48 h after cell attachment for subsequent analysis. Several key DEGs were selected and validated by real-time qPCR. The mRNA expressions of the core upregulated and downregulated genes were analyzed by real-time qPCR. Western blotting was conducted for further validation. Total RNA from cells was extracted using the Trizon reagent (CW0580S, CWBIO), and mRNA was extracted using an RNA Ultra-Pure Extraction Kit (CW0581M, CWBIO). Next, 2 µg of mRNA from each sample was reverse transcribed to single-stranded cDNA using the Reverse Transcription Kit HiScript II Q RT SuperMix for qPCR (R223-01, Vazyme) to synthesize cDNA, and fluorescence PCR was performed using a fluorescence PCR instrument (CFX Connect™ real-time, BoLe Life Medical Products Shanghai Co., Ltd.). One microliter of cDNA was used as a template for the following PCR. The quantitative PCR comprised an initial denaturation at 95°C for 35 s, then 40 cycles at 95°C for 10 s plus 58°C for 30 s, and lastly, extension at 72°C for 30 s. β-actin was used as an internal reference. The sequences of the primers are listed in the supplemental material. Relative quantification of mRNA expression was calculated using the 2^−ΔΔCT^ method. All samples were examined in triplicate.

### 2.9 The HCM animal model

Male C57BL/6 mice (8 weeks old, SiBeiFu, Beijing, China) were housed at 20–26°C, 40%–70% humidity, and 12/12 h light/dark conditions. Modeling was initiated after 1 week of adaptation. Each animal was injected subcutaneously with ISO (30 mg/kg) in the back of the neck once per day for 2 weeks. In the 3rd week, the mice were injected every other day for another 2 weeks. In addition, for the therapy groups, intraperitoneal injections of the drugs were administered at the same time (once per day for 2 consecutive weeks and every other day starting in the 3rd week). Ultrasonography was performed after the 4 weeks of modeling, and special attention was paid to the following features: fraction of shortening (FS), ejection fraction (EF), cardiac output (CO), and stroke volume (SV) of the left ventricle. Finally, the extent of myocardial hypertrophy was assessed by hematoxylin and eosin (H&E) staining to measure the cross-sectional area (CSA) of the cardiomyocytes.

### 2.10 The therapeutic role of potential drugs in the HCM animal model

Based on drug sensitivity prediction and miRNA matching in bioinformatics analysis, the following drugs (including miRNAs) may have therapeutic effects: miR-1469, acetaminophen, propylthiouracil, methapyrilene, triptolide, and tretinoin (retinoic acid). Thereby, animals were divided into the following groups: model, model + miR-1469 (10 nmol/mouse), model + acetaminophen (150 mg/kg), model + propylthiouracil (16 mg/kg), model + methapyrilene (10 mg/kg), model + triptolide (100 ug/kg), and model + retinoic acid (20 mg/kg). The therapeutic roles of potential drugs were first assessed by ultrasonography results and then by histological analysis, including HE staining, of the heart tissue. The dosages of each drug (or the miRNA mimic) are listed in the supplemental material.

### 2.11 Immunohistochemical analysis of the validated key DEGs

For the changed key DEGs (*ATRNL1* and *WNT9A*) in the vitro cell model, immunohistochemical (IHC) staining was performed based on the tendency of animal heart tissue to show the impact of drugs on the protein expression. Paraffin sections of cardiac tissues were dewaxed and hydrated. Then, sections were treated with citrate buffer and freshly prepared in 3% hydrogen peroxide, followed by sealing with bovine serum albumin (BSA) (Solarbio) for 30 min at 37°C. The sections were incubated with rabbit anti-*ATRNL1* (bs-11504R, Bioss, 1/200) or rabbit anti-*WNT9A* (bs-1933R, Bioss, 1/200) overnight at 4°C. After washing, horseradish enzyme-labeled goat anti-rabbit IgG (H + L) (ZB-2301, Nakasugi Jinqiao, 1/100) was added dropwise and incubated at 37°C for 30 min. After re-staining with hematoxylin, the slices were blocked and observed under the microscope (CX43, OLYMPUS).

## 3 Results

### 3.1 DEGs in HCM

**FIGURE 1:**
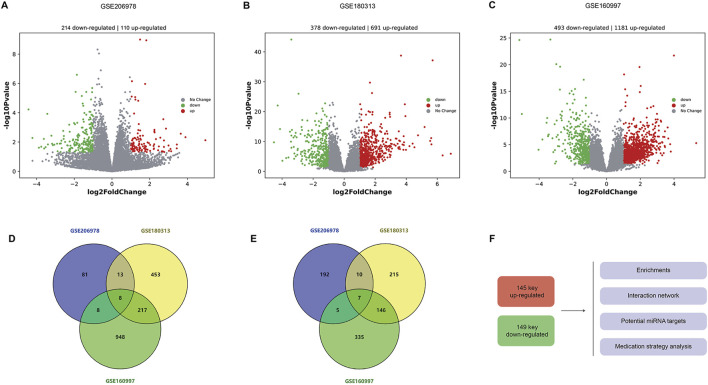
Differentially expressed genes in HCM. **(A)** Volcanic plots of DEGs in the GSE206978 dataset, with 214 upregulated and 110 downregulated genes. **(B)** Volcano plots of DEGs in the GSE180313 dataset, with 691 upregulated and 378 downregulated genes. **(C)** Volcanic plots of DEGs in the GSE160997 dataset, with 1181 upregulated and 493 downregulated genes. **(D)** Intersections of upregulated genes: eight common DEGs are shared by shared by three subsets. **(E)** Intersections of downregulated genes, with seven core downregulated genes. **(F)** These 145 key upregulated genes (with known functions and symbols) and 149 key downregulated genes (with known functions and symbols) are sent for further analysis, including enrichments, interaction networks, and consistent miRNA–mRNA matches, and potential drugs are predicted for HCM (using the core DEGs).

In the GSE206978 dataset, 214 upregulated and 110 downregulated genes are observed, as the volcano plots show (Figure 1A). In the GSE180313 dataset, there are 691 upregulated and 378 downregulated genes (Figure 1B), and in the GSE160997 dataset, there are 1181 upregulated and 493 downregulated genes (Figure 1C). The intersections of upregulated genes were analyzed (Figure 1D): eight common DEGs (*ATRNL1*, *BANCR*, *CRADD-AS1*, *GSG1L*, *MED12L*, *PENK*, *SH3GL2*, and *WNT9A*) are shared by three subsets and are considered as core upregulated DEGs. Together, 246 upregulated DEGs are shared by any two sets, within which there are 145 known genes (with definite symbols). The intersections of the downregulated genes are shown in Figure 1E, with seven core downregulated genes (*CHGB*, *EPHB2*, *HAS2*, *LRRN3*, *OSR1*, *SFRP5*, and *SPOCK1*). Among three sets, there are overall 168 downregulated DEGs shared by any two sets, and 149 DEGs are known genes. These 145 key upregulated genes and 149 key downregulated genes were then sent for further analysis, including enrichments, interaction networks, consistent miRNA-mRNA matches, and potential drugs for HCM using the core DEGs (Figure 1F).

### 3.2 Enrichment analysis of key upregulated and downregulated genes

**FIGURE 2:**
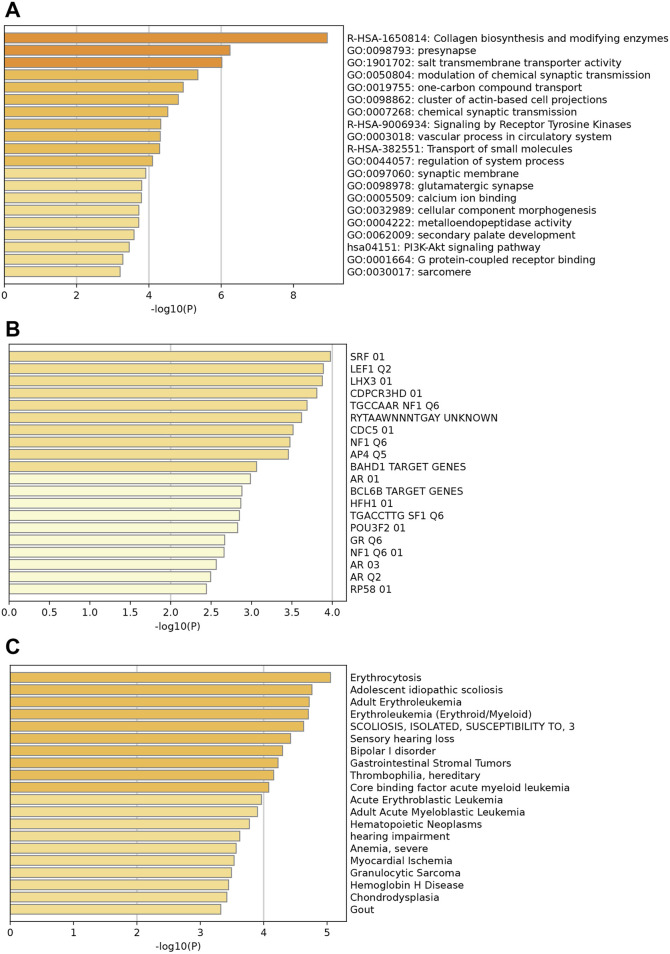
Enrichment analysis of key upregulated genes. **(A)** Top enriched GO terms/ KEGG pathways/Reactome terms. **(B)** The upregulated DEGs may be regulated by the top enriched transcription factors. **(C)** Enriched DisGeNET terms to show diseases associated with key upregulated genes.

Figure 2 illustrates the enrichments of key upregulated genes. Genes were mainly enriched in collagen biosynthesis and modifying enzymes, presynapse, and salt transmembrane transporter activity (Figure 2A). Additionally, prominent enriched reactome terms encompass the transport of small molecules, signaling by receptor tyrosine kinases, and collagen biosynthesis, among others. The enriched KEGG pathways feature the PI3K-Akt signaling pathway, protein digestion and absorption, and various others related to cellular and molecular processes. Figure 2B indicates that these upregulated differentially expressed genes (DEGs) may be regulated by enriched transcription factors such as *SRF*, *LEF1*, *LHX3*, and others. Moreover, associated diseases with these key upregulated genes, shown in Figure 2C, include erythrocytosis, adolescent idiopathic scoliosis, and several others; these conditions may increase the likelihood of HCM development and should be clinically noted.

**FIGURE 3:**
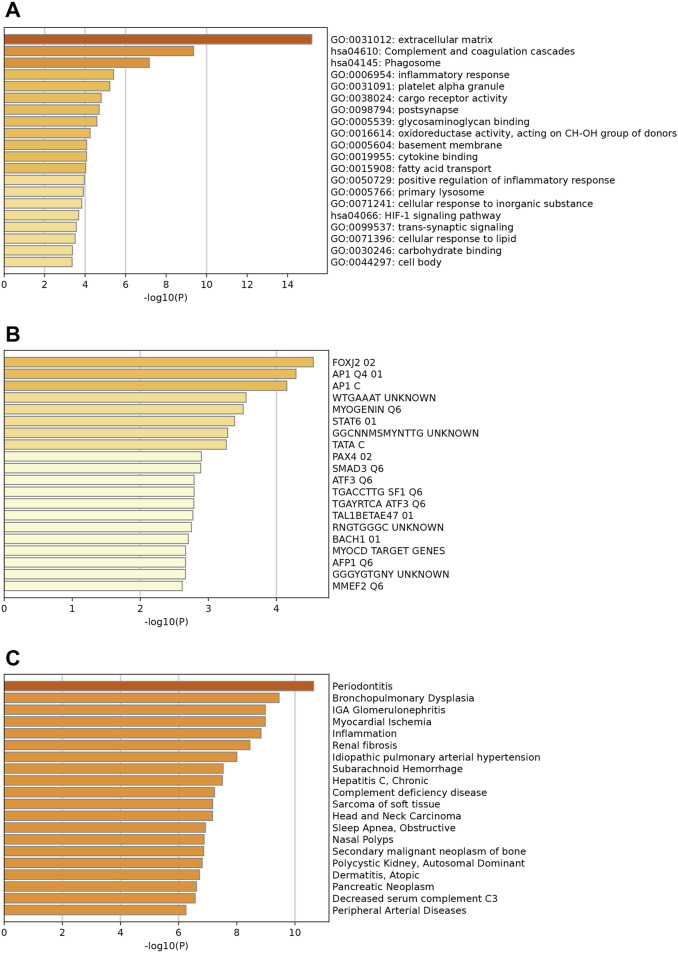
Enrichment analysis of key downregulated genes. **(A)** Top enriched GO terms/ KEGG pathways/Reactome terms. **(B)** The downregulated DEGs may be regulated by the top enriched transcription factors. **(C)** Enriched DisGeNET terms to show diseases associated with key downregulated genes.

The enrichment analysis for downregulated genes shows key GO terms such as extracellular matrix, inflammatory response, and innate immune response (Figure 3A). Significant reactome terms include hemostasis, metabolism of lipids, and neutrophil degranulation. Major enriched KEGG pathways are the complement and coagulation cascades, the phagosome, and alcoholic liver disease. The transcription factors involved are *FOXJ2*, *AP1*, and *MYOGENIN* (Figure 3B). Associated diseases, including Periodontitis, Myocardial Ischemia, and Renal fibrosis, are highlighted by enriched DisGeNET terms, which illustrate the relevance of these conditions related to the key down-regulated genes (Figure 3C).

**FIGURE 4:**
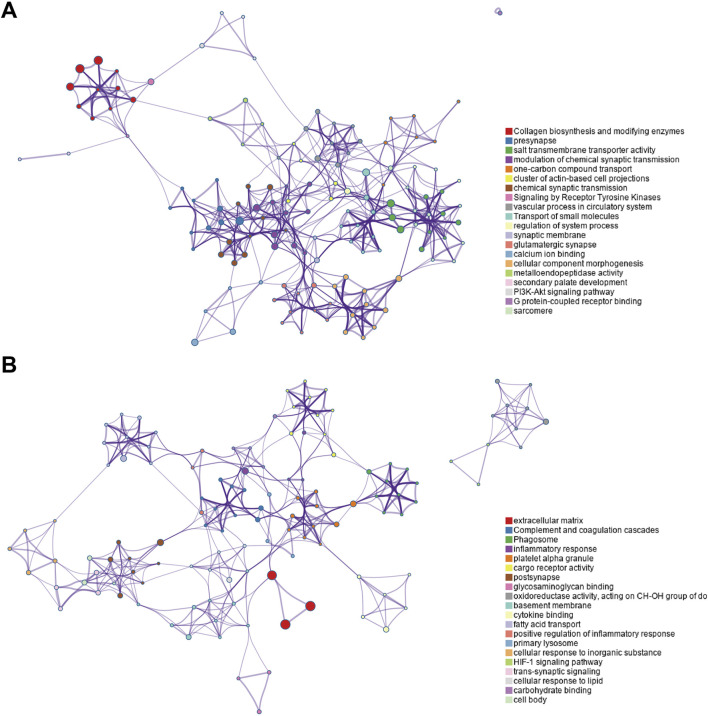
Enriched GO/KEGG/Reactome networks of key upregulated and downregulated genes. **(A)** Networks of key upregulated genes. **(B)** Networks of downregulated genes.

The enriched GO/KEGG/Reactome networks of key upregulated and downregulated genes are shown in Figures 4A, B.

### 3.3 Key gene PPI networks

**FIGURE 5:**
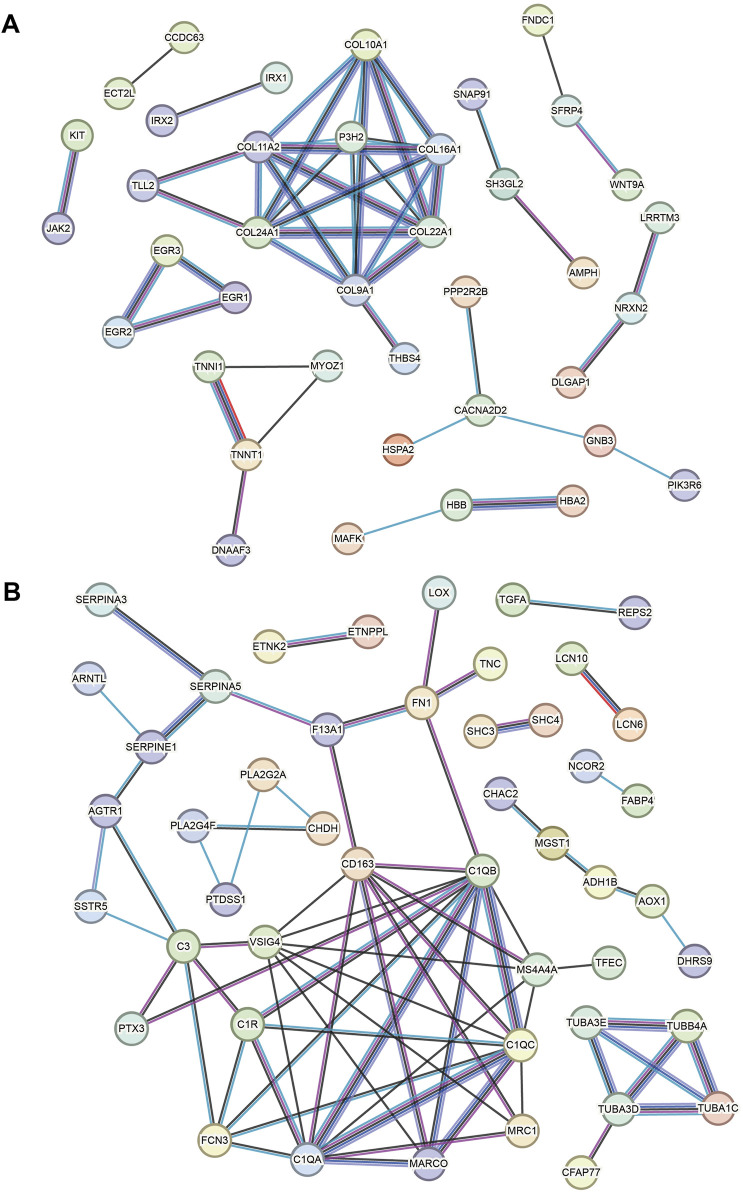
Key gene PPI networks. **(A)** PPI network based on the key upregulated genes. **(B)** PPI network based on the downregulated genes.

Based on the key upregulated and downregulated genes, interaction networks are shown in Figures 5A, B (using the STRING database). In the upregulated DEG network, *COL11A1*, *COL11A2*, *COL24A1*, *COL9A1*, and *P3H2* are located at the hub position (Figure 5A). In the downregulated DEG network, the following are import hub genes: *C1QB*, *C1QC*, *C1QA*, *VSIG4*, and *CD163* (Figure 5B).

### 3.4 Consistent miRNA–mRNA matches

**FIGURE 6:**
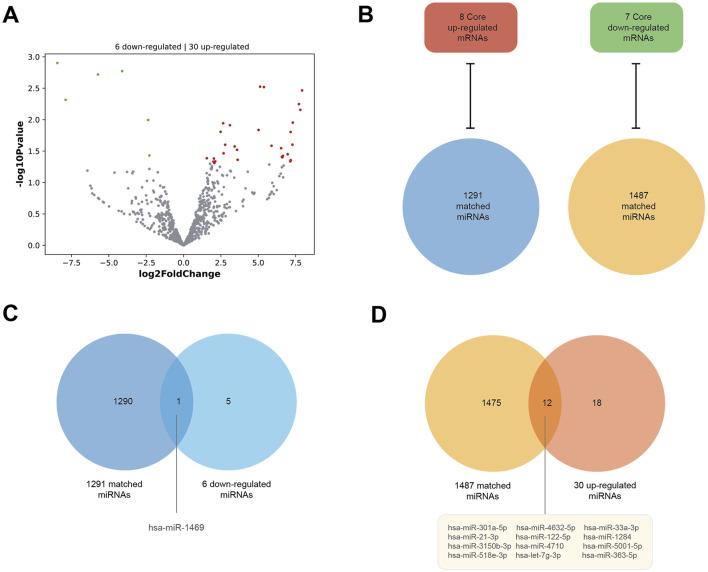
Consistent miRNA–mRNA matches. **(A)** DE miRNAs of HCM in the GSE197191 dataset. There are 30 upregulated and six downregulated miRNAs. **(B)** The miRWalk database shows 1291 miRNAs matched against the eight core upregulated mRNAs (they are theoretically downregulated), and 1487 miRNAs matched against the seven core downregulated mRNAs (theoretically upregulated in expression). **(C)** There is one miRNA, hsa-miR-1469, between the six downregulated miRNAs in the GSE197191 dataset and the 1291 matched miRNAs. **(D)** There are 12 miRNAs between the 30 upregulated miRNAs in the GSE197191 dataset and the 1487 matched miRNAs.

As Figure 6A shows, the DE miRNAs of HCM were analyzed in parallel using the GSE197191 dataset. There are 30 upregulated and six downregulated miRNAs. Meanwhile, using the miRWalk database, we obtained 1291 matched miRNAs against the eight core upregulated mRNAs (they are theoretically downregulated) and 1487 matched miRNAs against the seven core downregulated mRNAs (theoretically upregulated in expression) (Figure 6B). Between the six downregulated miRNAs in GSE197191 and the 1291 matched miRNAs, there is only one common miRNA, has-miR-1469 (Figure 6C). Similarly, between the 30 upregulated miRNAs in GSE197191 and the 1487 matched miRNA, there are 12 common miRNAs (Figure 6D). In future studies, modulating the expression of these miRNA targets could be a potentially novel means of HCM treatment.

### 3.5 Potential drugs for HCM based on core DEGs

**FIGURE 7:**
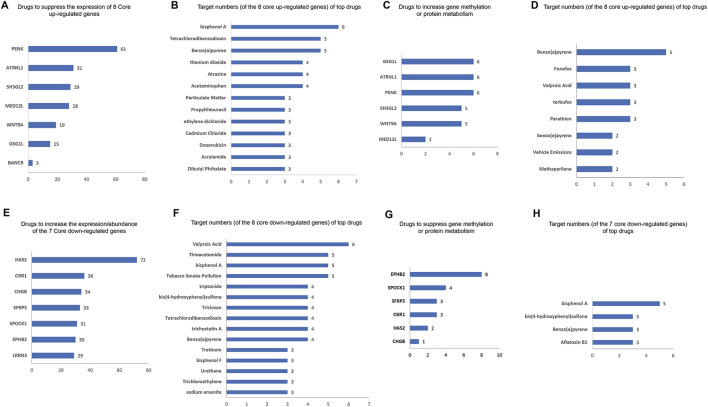
Potential drugs for HCM based on core DEGs. **(A)** Numbers of potential inhibitors of the eight core upregulated DEGs. **(B)** Of the eight core upregulated DEGs, the top-ranked inhibitors (ranked by most potential targets) are shown. **(C)** Number of drugs that increase the methylation or enhance the metabolism of the eight core upregulated DEGs. **(D)** Top-ranked drugs that increase the methylation or enhance the metabolism of the eight core DEGs. **(E)** Number of potential enhancers of the seven core downregulated DEGs (increasing their expression). **(F)** Of the seven core downregulated DEGs, the top potential enhancers (ranked by most potential targets) are shown. **(G)** Number of drugs inhibiting the methylation or metabolism of seven core downregulated DEGs. **(H)** Of the seven core downregulated DEGs, the top-ranked drugs that inhibit their methylation or metabolism are shown.

We identified the drugs that impact the expression/abundance/methylation/metabolic process of core DEGs using the CTD database. The potential inhibitors to the eight core upregulated DEGs are shown in Figure 7A. *PENK* has the most potential inhibitors (61), followed by *ATRNL1* (31 inhibitors), *SH3GL2* (29 inhibitors), and *MED12L* (28 inhibitors). The top inhibitors (ranked by most potential targets) of the eight core DEGs are shown in Figure 7B. Excluding significant toxic ingredients, acetaminophen and propylthiouracil are potential medications for HCM. The number of drugs that might increase the methylation or enhance the metabolism of eight core upregulated DEGs are shown in Figure 7C. The top drugs (modulating the highest number of core DEGs) are shown in Figure 7D. All drugs except methapyrilene (an oral H1 receptor antihistamine and pyridine anticholinergics) are toxic. The number of drugs that can increase the expression) of the seven core downregulated DEGs are shown in Figure 7E. The top drugs (excluding the clearly toxic ones) include triptonide, trichostatin A, and tretinoin (Figure 7F). The numbers of drugs that could inhibit the methylation or metabolism of the seven core downregulated DEGs are shown in Figure 7G. The top drugs all have definite toxicity and are not for human use (Figure 7H). Together, according to the modulation of DEGs, acetaminophen, propylthiouracil, methapyrilene, triptonide, trichostatin A, and tretinoin are potential medications for HCM treatment.

### 3.6 Validation of key gene expression in cell models

**FIGURE 8:**
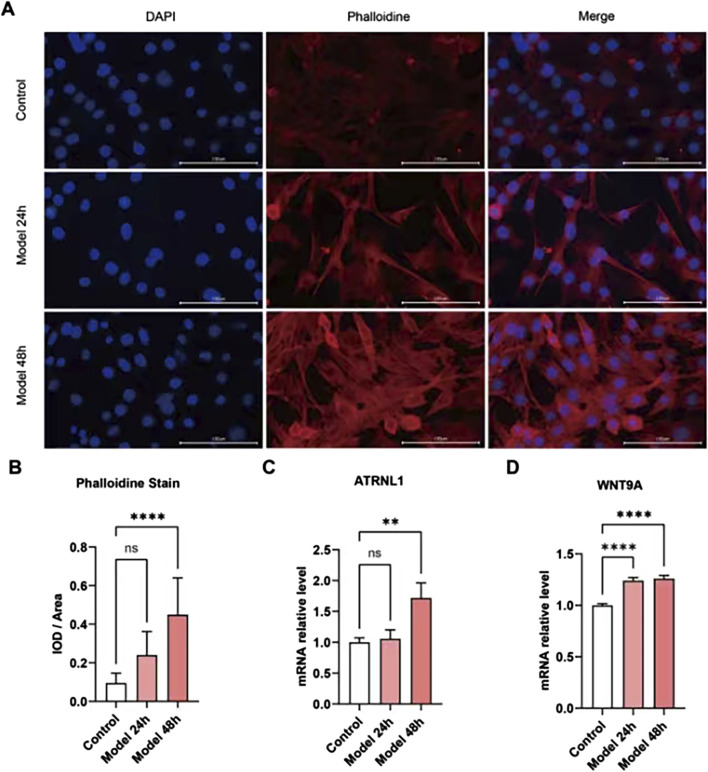
Validation of key gene expression in cell models. **(A)** Phalloidin immunofluorescence staining of the HCM cell model established by ISO. **(B)** The integrated optical density (IOD) of phalloidin is significantly increased at 48 h. **(C)** *ATRNL1* (mRNA) expression is significantly increased at 48 h. **(D)** *WNT9A* is significantly increased at 24 h and 48 h.

Changes in the core DEGs were detected in the HCM cell model. Given that many of these genes have been studied in HCM, we analyzed the expression of *ATRNL1*, *BANCR*, *GSG1L*, *MED12L*, *SH3GL2*, *WNT9A*, *CHGB*, and *LRRN3*, which have not been clearly reported in the HCM field. Meanwhile, among key nodes in the PPI network, *P3H2*, *CD163*, and *VSIG4* are novel genes in HCM. The expressions of the above genes in the HCM model cells were analyzed at 24 h and 48 h using real-time qPCR. As Figures 8A, B show, the HCM model was successfully established because the integrated optical density (IOD) of the phalloidin staining was significantly increased at 48 h. Among all the potential key DEGs, only *ATRNL1* (Figure 8C) and *WNT9A* (Figure 8D) were significantly changed (upregulated, in line with the bioinformatics analysis above) at 48 h.

### 3.7 Validation of drug efficacy in the HCM animal model

**FIGURE 9:**
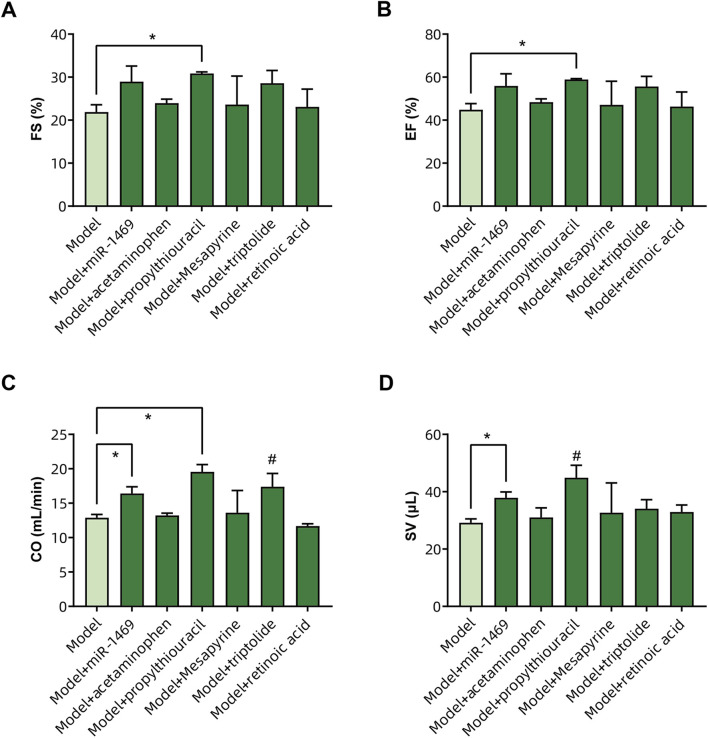
Validation of drug efficacy in the HCM animal model. The impacts of different drugs on the **(A)** fraction of shortening (FS%), **(B)** ejection fraction (EF%), **(C)** cardiac output (CO), and **(D)** stroke volume (SV) of the left ventricle. ^*^ Significance by unpaired t-tests with Welch’s correction, # significance by uncorrected Fisher’s LSD.

**FIGURE 10:**
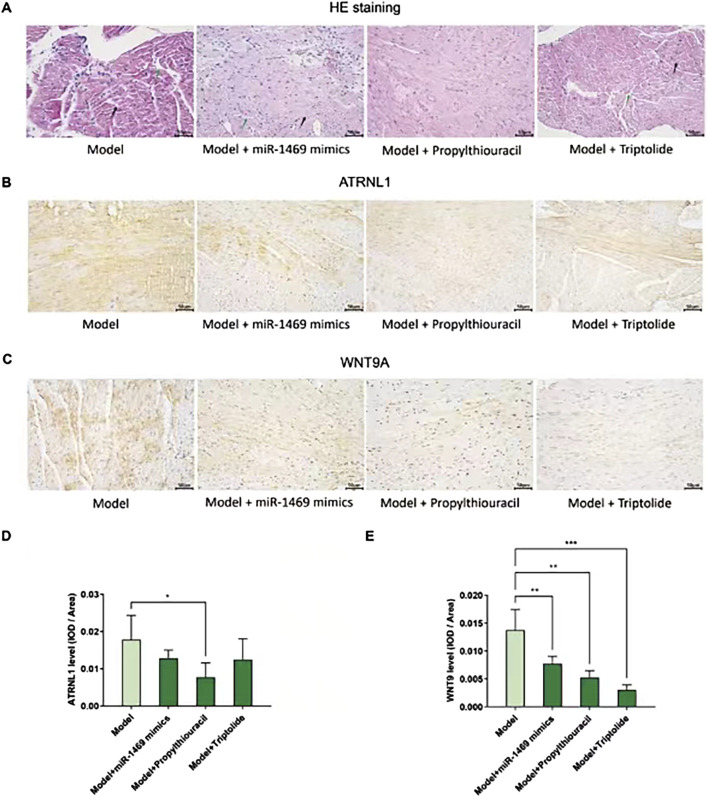
Pathological morphology and *ATRNL1/WNT9A* IHC staining in different groups. **(A)** HE stains of four groups: model, model + miR-1469, model + propylthiouracil, and model + triptolide. The model + miR-1469 and model + propylthiouracil groups showed a slight improvement of abnormalities in myocardial tissues. **(B)** IHC staining of *ATRNL1*. **(C)** IHC staining of *WNT9A*. **(D)** Only propylthiouracil, but not miR-1469 or triptolide, significantly inhibits the expression of *ATRNL1* in the HCM model. **(E)** All of the three drugs suppress *WNT9A* expression. ^*^ *p* < 0.05, ^**^ *p* < 0.01, and ^***^ *p* < 0.001.

Among five potential drugs and the miR-1469 mimic (miR-1469), only propylthiouracil showed a consistent therapeutic effect on various ultrasound indicators, including FS% (Figure 9A), EF% (Figure 9B), CO (Figure 9C), and SV (Figure 9D, using uncorrected Fisher’s LSD instead of an unpaired t-test with Welch’s correction). In addition, miR-1469 (by the method of an unpaired t-test with Welch’s correction) and triptolide (using the uncorrected Fisher’s LSD method) also increased CO (Figure 9C), and miR-1469 simultaneously improved SV (Figure 9D, according to the method of an unpaired t-test with Welch’s correction). Together, propylthiouracil, miR-1469, and triptolide demonstrated varying degrees of therapeutic effects on HCM. Therefore, we observed the pathological morphology of the following four groups: model, model + miR-1469, model + propylthiouracil, and model + triptolide. Indeed, the model + miR-1469 and model + propylthiouracil groups showed a slight improvement of abnormalities in myocardial tissues (Figure 10A).

### 3.8 The impact of drugs on ATRNL1 and WNT9A expression in the HCM animal model

Given the therapeutic effects of propylthiouracil, miR-1469, and triptolide on HCM, the protein expressions of *ATRNL1* (Figure 10B) and *WNT9A* (Figure 10C) were analyzed using IHC staining. Only propylthiouracil, but not miR-1469 or triptolide, significantly inhibited the expression of *ATRNL1* in the HCM model (Figure 10D), and all three drugs suppressed *WNT9A* expression (Figure 10E). This result not only confirmed that the three drugs may indeed exert anti-HCM effects but also further validated that overexpression of *ATRNL1* and *WNT9A* may be involved in HCM development.

## 4 Discussion

In this study, we discovered 145 key upregulated and 149 downregulated DEGs associated with HCM development, among which there are eight core upregulated genes (*ATRNL1*, *BANCR*, *CRADD-AS1*, *GSG1L*, *MED12L*, *PENK*, *SH3GL2*, and *WNT9A*) and seven core downregulated genes (*CHGB*, *EPHB2*, *HAS2*, *LRRN3*, *OSR1*, *SFRP5*, and *SPOCK1*). Additionally, we have found several hub genes that are particularly crucial for HCM, including *COL11A1*, *COL11A2*, *COL24A1*, *COL9A1*, *P3H2*, *C1QB*, *C1QC*, *C1QA*, *VSIG4*, and *CD163*. The lack of miR-1469 may contribute to the development of HCM, and its effectiveness as a therapy should be investigated in future research. Based on the modulation of DEGs, acetaminophen, propylthiouracil, methapyrilene, triptonide, trichostatin A, and tretinoin have shown potential as medications for treating HCM. In addition, through cell and animal experiments, we further confirmed that the high expression of *ATRNL1* or *WNT9A* was indeed involved in the development of HCM. miR-1469, propylthiouracil, and tretinoin could counteract the development of HCM to different degrees; they could also regulate the expression of *ATRNL1* and *WNT9A*.

Among the core DEGs, *PENK* is a known risk for hypoxia and immune-related conditions associated with HCM (Ahmadi et al., 2022; Zheng et al., 2021; Yu et al., 2023). It may be involved in the circRNA/miRNA/mRNA network in the pathogenesis of recurrent implantation failure associated with HCM (Ahmadi et al., 2022). However, its specific pathogenic mechanism in HCM remains unclear. Inhibition of *EPHB2* in HCM can be supported by related studies. Developmental *SHP2* dysfunction underlies cardiac hypertrophy in Noonan syndrome partially through the decreased *NOTCH1/EPHB2* signaling (Lauriol et al., 2016). Meanwhile, a related mechanism of its deficiency underlying HCM may be the inhibition of *RIT1* signals (Cavé et al., 2016). Previously, it has been mentioned that *HAS2* may be decreased in HCM (Lorén et al., 2019), even though detailed evidence is still scarce. In a bioinformatics study, *OSR1* is a significantly decreased DEG, which is in line with our finding (Wu et al., 2022), and similar but still weak evidence showed that *SFRP5* and *SPOCK1* may be involved in the pathology of HCM (Liu et al., 2016; Ma et al., 2021). However, there have been currently no direct links between HCM and *ATRNL1*, *BANCR*, *GSG1L*, *MED12L*, *SH3GL2*, *WNT9A*, *CHGB*, or *LRRN3*.

In this study, for the first time, we confirmed the involvement of *ATRNL1* and *WNT9A* in the pathogenesis of HCM in combination with bioinformatics analysis and experiments. For *ATRNL1* (attractin like 1), known research is still very limited. GO annotations related to this gene include signaling receptor activity and carbohydrate binding. In addition, *ATRNL1* is associated with the development of some tumors (Ding et al., 2024; Robesova et al., 2015; Fang et al., 2023), suggesting that its function has the potential to promote cell growth, which may constitute one of the mechanisms of HCM. However, *ATRNL1* is mainly distributed in the cell membrane, and more specific downstream mechanisms remain to be elucidated. *WNT9A* is a member of the WNT family. Among its related pathways are signaling by *WNT* and Nanog in mammalian ESC pluripotency. In zebrafish, WNT9A and the downstream Wnt signaling regulate the migration of cardiomyocyte progenitor cells and control the formation of the cardiac tube (Paolini et al., 2023). This can be partly attributed to their regulation of the timing of cardiac progenitor cell differentiation, and Wnt9-triggered *WNT* signaling activation may constitute one of the pathogenic mechanisms of HCM. Our results showed that the expression of *ATRNL1* and *WNT9A* are elevated in HCM cellular and animal models and decreased with improvement of HCM after drug treatment, suggesting that ATRNL1 and WNT9A are indeed important targets of HCM pathogenesis.

In the PPI network, multiple collagen-related genes are important nodes for key DEGs in HCM. This finding is reasonable, for myocardial collagen alteration/turnover/remodeling is a key pathogenic mechanism of HCM (Lombardi et al., 2003; Kitamura et al., 2001). Myocardial interstitial fibrosis is a characteristic of HCM, underlying which collagen turnover is enhanced in HCM patients (Lombardi et al., 2003). Peripheral levels of byproducts of collagen synthesis reflect myocardial extracellular matrix metabolism in HCM patients (Ellims et al., 2014). The collagen volume fraction has prognostic value in HCM patients (Arteaga et al., 2009). The ECM proteoglycan lumican is increased in HCM and colocalizes with fibrillar collagen throughout areas of fibrosis (Rixon et al., 2023). Moreover, collagen cross-linking is associated with cardiac remodeling in hypertrophic obstructive HCM (Bi et al., 2021).

In addition to collagen genes, the complement C1q genes (*C1QB*, *C1QC*, and *C1QA*) are hub genes in our network result. Similar results have been reported in a bioinformatics work [15]. Consistent results have been reported in associated studies (Li et al., 2023), but the mechanism of the action of complement C1q in HCM has not been understood. Furthermore, it has been confirmed to be associated with mitochondrial cardiomyopathy (Wang et al., 2022). However, these results suggest that the development of HCM may be associated with immune cell abnormalities and that peripheral blood levels of complement C1q may be useful in HCM monitoring. *VSIG4* (V-set and immunoglobulin domain-containing 4, also known as the Complement receptor of the immunoglobulin superfamily) is a membrane receptor associated with the complement system that is expressed in many different cell types, including macrophages, dendritic cells, neutrophils, and hepatocytes, and is involved in a wide variety of biological processes including immune response and inflammatory regulation. *VSIG4* has been known as a significantly decreased gene in HCM (Wu et al., 2022). Among hub genes, *CD163* is a type-I membrane protein that is predominantly expressed in the monocyte/macrophage system. Together with complement *C1q* and *VSIG4*, our PPI results implied the role of immune cells in the pathogenesis of HCM, and this has also been noted in previous studies (Zhang et al., 2021). Finally, *P3H2* is a very new gene in HCM, and its role is still to be investigated.

In our analysis of potential drugs, we proposed that the following drugs may be beneficial in HCM treatment: acetaminophen, propylthiouracil, methapyrilene, triptolide, trichostatin A, and tretinoin. There is no routine use of acetaminophen for HCM, and cases of its use in HCM are limited. There have been case reports of combined acetaminophen use in Noonan syndrome (Takagi et al., 2022). There is indeed evidence that patients with HCM can safely use acetaminophen for other reasons (Al Yazidi et al., 2022; Malik et al., 2022). For the first time, we here propose that acetaminophen may treat HCM through inhibition of four core upregulated DEGs: *GSG1L*, *MED12L*, *PENK*, and *SH3GL2*. This novel mechanism and potential effect need to be validated on a priority basis. Propylthiouracil reduces thyroid hormone synthesis by inhibiting the peroxidase system in the thyroid gland. It also has immunosuppressive effects. Considering the above results about key DEGs and hub DEGs suggests that immune activation may be involved in the development of HCM. It is expected that propylthiouracil can play a role in the treatment of HCM. There are cases of propylthiouracil usage in HCM (Lowey et al., 2008), although at this time, the hypothesis is very novel, and no definitive studies have confirmed its role. However, in animal studies, expression of β/slow MHC in the heart may be induced by administering propylthiouracil (Fielitz et al., 2007). Therefore, its clinical application is still premature, and animal experiments are needed to verify its efficacy. Here, we demonstrated for the first time that propylthiouracil can improve the ultrasound indicators and pathomorphology of HCM model animals, and this result provides an important reference for the pharmacological treatment of HCM. The therapeutic effect of propylthiouracil was more reliable and consistent than that of other drugs, with positive results in several indicators. In particular, propylthiouracil should be preferred in HCM patients with hyperthyroidism. Moreover, according to the CTD database, propylthiouracil can inhibit the expression of both *ATRNL1* and *WNT9A*. This function was validated in our animal model. Thus, we have not only demonstrated for the first time the therapeutic efficacy of propylthiouracil against HCM in the animal model but also revealed for the first time the underlying molecular mechanism of its efficacy (by weakening *ATRNL1* and *WNT9A* signaling).

Regarding trichostatin A, one study demonstrated the different responses of a genetic HCM zebrafish model to trichostatin A and U0126 (Becker et al., 2012). Then, in 2014, a study used patient-specific induced pluripotent stem cell (iPSC) derived cardiomyocytes as the HCM model and tested whether trichostatin A could prevent the development of disease phenotypes in HCM iPSC-cardiomyocytes (Han et al., 2014). The results showed that continuous administration of trichostatin A improved the hypertrophic phenotypes of HCM cells, including decreased size and suppressed *NFATC* nuclear translocation. In addition, CK2α1-transgenic mice can develop HCM, which was attenuated by the administration of trichostatin A (Eom et al., 2011). These results confirmed our hypothesis, and we here provide a new underlying mechanism that trichostatin A can simultaneously elevate four core downregulated DEGs: *CHGB, HAS2, LRRN3, and SPOCK1*.

To date, no studies have reported the link between methapyrilene, triptolide, or tretinoin and HCM. Our animal experiments suggest that triptolide can be an adjunctive agent for HCM. This result is consistent with research on other cardiomyopathies. For example, triptolide can be used as an anti-diabetic-cardiomyopathy agent (Zhu et al., 2021; Wen et al., 2013; Liang et al., 2015).

Lastly, we found that the miR-1469 mimic is also a useful anti-HCM agent. The miR-1469 mimic reduced *WNT9A* expression while improving HCM. This result is consistent with our expectations. In the results of bioinformatics analysis, miR-1469 is a downregulated RNA in HCM, and it is also one of the matched miRNAs against eight core upregulated DEGs. In the miRWalk database, *WNT9A* is the target (3UTR) of miR-1469 (Score = 1, energy = −32.2, 15 pairings). The above results suggest that downregulation of miR-1469 may lead to increased *WNT9A* expression and contribute to the development of HCM.

Still, this study has some limitations. Although we have obtained some consistent correlation results through cell and animal experiments, we have not yet validated the molecular mechanisms of *ATRNL1* and *WNT9A* in HCM development and treatment by knockdown methods. In addition, due to the limited sample size, many results have not yet reached significant differences, and further subsequent validation is urgently needed.

## Data Availability

The datasets presented in this study can be found in online repositories. The names of the repository/repositories and accession number(s) can be found in the article/supplementary material.

## References

[B1] Ahmadi M. Pashangzadeh S. Moraghebi M. Sabetian S. Shekari M. Eini F. (2022). Construction of circRNA-miRNA-mRNA network in the pathogenesis of recurrent implantation failure using integrated bioinformatics study. J. Cell Mol. Med. 26 (6), 1853–1864. 10.1111/jcmm.16586 33960101 PMC8918409

[B2] Al Yazidi G. Mulder J. Licht C. Harvey E. Robertson J. Sondheimer N. (2022). Reversal of stroke-like episodes with L-arginine and meticulous perioperative man-agement of renal transplantation in a patient with mitochondrial encephalomyopathy, lactic acidosis and stroke-like ep-isodes (MELAS) syndrome. Case Rep. Neurohospitalist 12 (1), 67–73. 10.1177/19418744211000512 PMC8689537 34950389

[B3] Arteaga E. de Araújo A. Q. Bernstein M. Ramires F. J. A. Ianni B. M. Fernandes F. (2009). Prognostic value of the collagen volume fraction in hypertrophic cardiomyopathy. Arq. Bras. Cardiol. 92 (3), 210–214. 10.1590/s0066-782x2009000300010 19390710

[B4] Becker J. R. Robinson T. Y. Sachidanandan C. Kelly A. E. Coy S. Peterson R. T. (2012). *In vivo* natriuretic peptide reporter assay identifies chemical modifiers of hypertrophic cardiomyopathy signalling. Cardiovasc Res. 93 (3), 463–470. 10.1093/cvr/cvr350 22198505 PMC3410427

[B5] Bi X. Song Y. Song Y. Yuan J. Cui J. Zhao S. (2021). Collagen cross-linking is associated with cardiac remodeling in hypertrophic obstructive car-diomyopathy. J. Am. Heart Assoc. 10 (1), e017752. 10.1161/JAHA.120.017752 33356379 PMC7955480

[B6] Cavé H. Caye A. Ghedira N. Capri Y. Pouvreau N. Fillot N. (2016). Mutations in RIT1 cause Noonan syndrome with possible juvenile myelomonocytic leukemia but are not involved in acute lymphoblastic leukemia. Eur. J. Hum. Genet. 24 (8), 1124–1131. 10.1038/ejhg.2015.273 26757980 PMC4970687

[B7] Ding B. Ye Z. Yin H. Hong X. Y. Feng S. W. Xu J. Y. (2024). Comprehensive single-cell analysis reveals heterogeneity of fibroblast subpopulations in ovarian cancer tissue microenvironment. Heliyon 10 (6), e27873. 10.1016/j.heliyon.2024.e27873 38533040 PMC10963331

[B8] Ellims A. H. Taylor A. J. Mariani J. A. Ling L. H. Iles L. M. Maeder M. T. (2014). Evaluating the utility of circulating biomarkers of collagen synthesis in hypertrophic cardiomyopathy. Circ. Heart Fail 7 (2), 271–278. 10.1161/CIRCHEARTFAILURE.113.000665 24481111

[B9] Eom G. H. Cho Y. K. Ko J. H. Shin S. Choe N. Kim Y. (2011). Casein kinase-2α1 induces hypertrophic response by phosphorylation of histone deacetylase 2 S394 and its activation in the heart. Circulation 123 (21), 2392–2403. 10.1161/CIRCULATIONAHA.110.003665 21576649

[B10] Fang M. Zhou Y. Li Z. Li X. (2023). Transcriptional factor CCAAT enhancer binding protein beta inhibits epithelial-mesenchymal transition in cervical cancer via regulating attractin-like 1. Cell Mol. Biol. (Noisy-le-grand) 69 (13), 1–7. 10.14715/cmb/2023.69.13.1 38158696

[B11] Fielitz J. Kim M. S. Shelton J. M. Latif S. Spencer J. A. Glass D. J. (2007). Myosin accumulation and striated muscle myopathy result from the loss of muscle RING finger 1 and 3. J. Clin. Invest 117 (9), 2486–2495. 10.1172/JCI32827 17786241 PMC1957544

[B12] Geske J. B. Ommen S. R. Gersh B. J. (2018). Hypertrophic cardiomyopathy: clinical update. JACC Heart Fail 6 (5), 364–375. 10.1016/j.jchf.2018.02.010 29655825

[B13] Han L. Li Y. Tchao J. Kaplan A. D. Lin B. Li Y. (2014). Study familial hypertrophic cardiomyopathy using patient-specific induced pluripotent stem cells. Cardiovasc Res. 104 (2), 258–269. 10.1093/cvr/cvu205 25209314 PMC4217687

[B14] Kitamura M. Shimizu M. Ino H. Okeie K. Yamaguchi M. Funjno N. (2001). Collagen remodeling and cardiac dysfunction in patients with hypertrophic cardio-myopathy: the significance of type III and VI collagens. Clin. Cardiol. 24 (4), 325–329. 10.1002/clc.4960240413 11303702 PMC6654813

[B15] Lauriol J. Cabrera J. R. Roy A. Keith K. Hough S. M. Damilano F. (2016). Developmental SHP2 dysfunction underlies cardiac hypertrophy in Noonan syndrome with multiple lentigines. J. Clin. Invest 126 (8), 2989–3005. 10.1172/JCI80396 27348588 PMC4966304

[B16] Li X. Zhang B. Ding W. Jia X. Han Z. Zhang L. (2023). Serum proteomic signatures in umbilical cord blood of preterm neonates delivered by women with gestational diabetes. Diabetes Metab. Syndr. Obes. 16, 1525–1539. 10.2147/DMSO.S406297 37260850 PMC10228520

[B17] Liang Z. Leo S. Wen H. Ouyang M. Jiang W. Yang K. (2015). Triptolide improves systolic function and myocardial energy metabolism of diabetic cardiomyopathy in streptozotocin-induced diabetic rats. BMC Cardiovasc Disord. 15, 42–196. 10.1186/s12872-015-0030-4 25967112 PMC4431461

[B18] Liu J. Jing L. Tu X. (2016). Weighted gene co-expression network analysis identifies specific modules and hub genes related to coronary artery disease. BMC Cardiovasc Disord. 16, 54. 10.1186/s12872-016-0217-3 26944061 PMC4779223

[B19] Lombardi R. Betocchi S. Losi M. A. Tocchetti C. G. Aversa M. Miranda M. (2003). Myocardial collagen turnover in hypertrophic cardiomyopathy. Circulation 108 (12), 1455–1460. 10.1161/01.CIR.0000090687.97972.10 12952838

[B20] Lorén C. E. Dahl C. P. Do L. Almaas V. M. Geiran O. R. Mörner S. (2019). Low molecular mass myocardial hyaluronan in human hypertrophic cardiomyopathy. Cells 8 (2), 97. 10.3390/cells8020097 30699940 PMC6406527

[B21] Lowey S. Lesko L. M. Rovner A. S. Hodges A. R. White S. L. Low R. B. (2008). Functional effects of the hypertrophic cardiomyopathy R403Q mutation are different in an alpha- or beta-myosin heavy chain backbone. J. Biol. Chem. 283 (29), 20579–20589. 10.1074/jbc.M800554200 18480046 PMC2459289

[B22] Ma Z. Wang X. Lv Q. Gong Y. Xia M. Zhuang L. (2021). Identification of underlying hub genes associated with hypertrophic cardiomyopathy by inte-grated bioinformatics analysis. Pharmgenomics Pers. Med. 14, 823–837. 10.2147/PGPM.S314880 34285551 PMC8285300

[B23] Malik F. I. Robertson L. A. Armas D. R. Robbie E. P. Osmukhina A. Xu D. (2022). A phase 1 dose-escalation study of the cardiac myosin inhibitor aficamten in healthy participants. JACC Basic Transl. Sci. 7 (8), 763–775. 10.1016/j.jacbts.2022.04.008 36061336 PMC9436819

[B24] Maron B. J. Desai M. Y. Nishimura R. A. Spirito P. Rakowski H. Towbin J. A. (2022b). Diagnosis and evaluation of hypertrophic cardiomyopathy: JACC state-of-the-art review. J. Am. Coll. Cardiol. 79 (4), 372–389. 10.1016/j.jacc.2021.12.002 35086660

[B25] Maron B. J. Desai M. Y. Nishimura R. A. Spirito P. Rakowski H. Towbin J. A. (2022c). Management of hypertrophic cardiomyopathy: JACC state-of-the-art review. J. Am. Coll. Cardiol. 79 (4), 390–414. 10.1016/j.jacc.2021.11.021 35086661

[B26] Maron B. J. Rowin E. J. Maron M. S. (2022a). Hypertrophic cardiomyopathy: new concepts and therapies. Annu. Rev. Med. 73, 363–375. 10.1146/annurev-med-042220-021539 35084989

[B27] Olivotto I. Oreziak A. Barriales-Villa R. Abraham T. P. Masri A. Garcia-Pavia P. (2020). Mavacamten for treatment of symptomatic obstructive hypertrophic cardiomy-opathy (EXPLORER-HCM): a randomised, double-blind, placebo-controlled, phase 3 trial. Lancet 396 (10253), 759–769. 10.1016/S0140-6736(20)31792-X 32871100

[B28] Paolini A. Sharipova D. Lange T. Abdelilah-Seyfried S. (2023). Wnt9 directs zebrafish heart tube assembly via a combination of ca-nonical and non-canonical pathway signaling. Development 150 (18), dev201707. 10.1242/dev.201707 37680191 PMC10560569

[B29] Rixon C. Andreassen K. Shen X. Erusappan P. M. Almaas V. M. Palmero S. (2023). Lumican accumulates with fibrillar collagen in fibrosis in hypertrophic cardiomyopathy. Esc. Heart Fail 10 (2), 858–871. 10.1002/ehf2.14234 36444917 PMC10053290

[B30] Robesova B. Bajerova M. Hausnerova J. Skrickova J. Tomiskova M. Dvorakova D. (2015). Identification of atypical ATRNL1 insertion to EML4-ALK fusion gene in NSCLC. Lung Cancer 87 (3), 318–320. 10.1016/j.lungcan.2015.01.002 25601488

[B31] Rowin E. J. Maron B. J. Haas T. S. Garberich R. F. Wang W. Link M. S. (2017). Hypertrophic cardiomyopathy with left ventricular apical aneurysm: implications for risk stratification and management. J. Am. Coll. Cardiol. 69 (7), 761–773. 10.1016/j.jacc.2016.11.063 28209216

[B32] Semsarian C. Ingles J. Maron M. S. Maron B. J. (2015). New perspectives on the prevalence of hypertrophic cardiomyopathy. J. Am. Coll. Cardiol. 65 (12), 1249–1254. 10.1016/j.jacc.2015.01.019 25814232

[B33] Takagi S. Ando S. Kono R. Oono Y. Nagasaka H. Kohase H. (2022). Methemoglobinemia induced by prilocaine in a child with noonan syndrome. Anesth. Prog. 69 (3), 25–29. 10.2344/anpr-69-02-01 36223191 PMC9552618

[B34] Tuohy C. V. Kaul S. Song H. K. Nazer B. Heitner S. B. (2020). Hypertrophic cardiomyopathy: the future of treatment. Eur. J. Heart Fail 22 (2), 228–240. 10.1002/ejhf.1715 31919938

[B35] Wang J. Huang C. L. Zhang Y. (2022). Complement C1q binding protein (C1QBP): physiological functions, mutation-associated mi-tochondrial cardiomyopathy and current disease models. Front. Cardiovasc Med. 9, 843853. 10.3389/fcvm.2022.843853 35310974 PMC8924301

[B36] Wen H. L. Liang Z. S. Zhang R. Yang K. (2013). Anti-inflammatory effects of triptolide improve left ventricular function in a rat model of diabetic cardiomyopathy. Cardiovasc Diabetol. 12, 50. 10.1186/1475-2840-12-50 23530831 PMC3617021

[B37] Wu S. Ud Din I. Sadiq F. M. Abdel-Maksoud M. A. Haris M. Mubarak A. (2022). Dysfunctional network of hub genes in hypertrophic cardiomyopathy patients. Am. J. Transl. Res. 14 (12), 8918–8933. 36628247 PMC9827312

[B38] Yu H. Gu L. Du L. Dong Z. Li Z. Yu M. (2023). Identification and analysis of key hypoxia- and immune-related genes in hypertrophic cardiomyopathy. Biol. Res. 56 (1), 45. 10.1186/s40659-023-00451-4 37559135 PMC10410988

[B39] Zhang X. Z. Zhang S. Tang T. T. Cheng X. (2021). Bioinformatics and immune infiltration analyses reveal the key pathway and immune cells in the pathogenesis of hypertrophic cardiomyopathy. Front. Cardiovasc Med. 8, 696321. 10.3389/fcvm.2021.696321 34497835 PMC8419431

[B40] Zheng X. Liu G. Huang R. (2021). Identification and verification of feature immune-related genes in patients with hypertrophic cardiomyopathy based on bioinformatics analyses. Front. Cardiovasc Med. 8, 752559. 10.3389/fcvm.2021.752559 34765659 PMC8577723

[B41] Zhu N. Huang B. Zhu L. Wang Y. (2021). Potential mechanisms of triptolide against diabetic cardiomyopathy based on network pharmacology analysis and molecular docking. J. Diabetes Res. 2021, 9944589. 10.1155/2021/9944589 34926700 PMC8672107

